# Language Impairment in Alzheimer’s Disease—Robust and Explainable Evidence for AD-Related Deterioration of Spontaneous Speech Through Multilingual Machine Learning

**DOI:** 10.3389/fnagi.2021.642033

**Published:** 2021-05-19

**Authors:** Hali Lindsay, Johannes Tröger, Alexandra König

**Affiliations:** ^1^German Research Center for Artificial Intelligence, DFKI GmbH, Saarbrücken, Germany; ^2^ki elements, Saarbrücken, Germany; ^3^Institut national de recherche en informatique et en automatique (INRIA), Stars Team, Sophia Antipolis, Valbonne, France; ^4^CoBteK (Cognition-Behavior-Technology) Lab, FRIS—University Côte d’azur, Nice, France

**Keywords:** Alzheimer’s disease, dementia, spontaneous speech, language impairment, picture description, natural language processing, explainability, multilingual machine learning

## Abstract

Alzheimer’s disease (AD) is a pervasive neurodegenerative disease that affects millions worldwide and is most prominently associated with broad cognitive decline, including language impairment. Picture description tasks are routinely used to monitor language impairment in AD. Due to the high amount of manual resources needed for an in-depth analysis of thereby-produced spontaneous speech, advanced natural language processing (NLP) combined with machine learning (ML) represents a promising opportunity. In this applied research field though, NLP and ML methodology do not necessarily ensure robust clinically actionable insights into cognitive language impairment in AD and additional precautions must be taken to ensure clinical-validity and generalizability of results. In this study, we add generalizability through multilingual feature statistics to computational approaches for the detection of language impairment in AD. We include 154 participants (78 healthy subjects, 76 patients with AD) from two different languages (106 English speaking and 47 French speaking). Each participant completed a picture description task, in addition to a battery of neuropsychological tests. Each response was recorded and manually transcribed. From this, task-specific, semantic, syntactic and paralinguistic features are extracted using NLP resources. Using inferential statistics, we determined language features, excluding task specific features, that are significant in both languages and therefore represent “generalizable” signs for cognitive language impairment in AD. In a second step, we evaluated all features as well as the generalizable ones for English, French and both languages in a binary discrimination ML scenario (AD vs. healthy) using a variety of classifiers. The generalizable language feature set outperforms the all language feature set in English, French and the multilingual scenarios. Semantic features are the most generalizable while paralinguistic features show no overlap between languages. The multilingual model shows an equal distribution of error in both English and French. By leveraging multilingual statistics combined with a theory-driven approach, we identify AD-related language impairment that generalizes beyond a single corpus or language to model language impairment as a clinically-relevant cognitive symptom. We find a primary impairment in semantics in addition to mild syntactic impairment, possibly confounded by additional impaired cognitive functions.

## Introduction

Alzheimer’s disease (AD) is a pervasive neurodegenerative disease that affect millions worldwide and is the most recognizable through its primarily cognitive syndrome—dementia. From 2008 to 2018, over 200 medical trials failed to develop a cure for AD dementia (Ferreira et al., 2018) emphasizing that early detection and intervention is still the best course for managing AD.

AD dementia is most prominently associated with heterogeneous and broad cognitive impairment; the typical and earliest-observable hallmarks are impaired memory and executive functions (Buckner, 2004). However, language impairments have been reported occurring in preclinical AD as well as mild, moderate, and severe AD dementia (Kempler, 1995; Klimova et al., 2015) possibly providing a window for screening, continuous monitoring and disease management that can help improve quality of life (Taler and Phillips, 2008; Le et al., 2011; Berisha et al., 2015; Klimova et al., 2015). As language is a pervasive aspect of daily living, language-based AD dementia assessment is ecologically valid and, from the patient perspective, one of the least intrusive ways to assess symptoms of AD dementia. This situates language impairment as an interesting behavioral biomarker from both a clinical and patient perspective (Ferris and Farlow, 2013).

Evidence for language impairment in AD dementia stems from studies using a variety of assessments ranging from structured, clinically-validated tasks to unstructured conversation (for an overview, see Szatloczki et al., 2015). An example of a structured task would be a naming task where a person is shown images on cards and asked to name the object. However, naming tasks do not represent the structure or nuance of natural language. In comparison, an unstructured clinical interview between a clinician and patient produces spontaneous speech in its full variance but is difficult and costly to compare and evaluate for minimal changes in cognition, including language, on a qualitative level. Therefore, many reported studies use a standardized experimental setup to elicit spontaneous speech from subjects; often, this is done by picture description tasks (for an overview, see Mueller et al., 2018). In the middle of this spectrum, the picture description task is a clinically-validated task where a patient is asked to describe a standardized picture. This produces spontaneous speech about an anticipated set of topics that is comparable among populations.

With an emphasis on available picture description data, AD detection has been a popular field for applied automatic speech processing and advanced natural language processing (NLP). The goal of such studies is to ultimately discriminate between a form of dementia and healthy control subjects (HC). In a fully automatic system, an audio recording is automatically transcribed with automatic speech recognition (ASR; König et al., 2015). This creates two sources of information from the file: (1) the sound recording; and (2) the text transcription. To model these sources of information, features are either implicitly represented (Orimaye et al., 2014) or explicitly engineered to automate clinical qualitative analysis (Fraser et al., 2016) and extracted from both components of the task. These features are then used to train supervised machine learning (ML) classifiers to discriminated conditions between a pathological patient group and healthy subjects (Yancheva et al., 2015; Yancheva and Rudzicz, 2016; Fraser et al., 2019).

These recent computational approaches represent significant advances for a better understanding of the AD dementia-related language impairment and including the technical challenge to efficiently assess spontaneous speech, but we argue that there are still multiple caveats. With advanced computational techniques and ML methods, there is an increased complexity added to understand the classifiers’ decisions and the entailed clinical assumptions. In other words, good ML performance alone does not necessarily entail clinical evidence for language impairment as a cognitive symptom in AD dementia. Additional methodological precautions must be taken to ensure that findings are clinically-valid, generalizable and do not over fit to a single corpus or language. Hence, limitations in current research have been attributed to lacking standardization and comparability between diagnostic settings as well as a growing gulf between how computational features actually model clinically-observable change (de la Fuente Garcia et al., 2020). The result being a lack of translation between NLP research and clinical application.

We state, that a major research gap is present between the clinical understanding of language impairment (as a neurocognitive function impairment) apparent in everyday spontaneous speech and recent NLP techniques used together with ML for speech-based classification of AD. To overcome this, we will: (1) investigate automatically extracted NLP features from spontaneous picture descriptions with respect to their ability in robustly capturing clinically valid AD-related language impairment; and (2) train robust ML models capturing cognitive language impairment in AD with afore-identified generalizable and explainable NLP features.

## Background

In order to model language impairment in AD, we first investigate which subprocess of language are impaired as defined by clinical literature. Language impairment in AD dementia is characterized by declining semantic and pragmatic processes and reduced syntactic complexity. Semantic processes refer to the meaning of language. A reduction in semantic processes is often indicated by difficulty finding a specific word, loss of comprehension, finding the incorrect word, using ambiguous referents, creating new words, and loss of verbal fluency. Pragmatic processes refer to adapting language to a specific situation. Pragmatic deficits can result in a person with AD dementia language impairment speaking too loudly, speaking at in appropriate times, repeating themselves or digressing from the topic. Syntactic processes are associated with the underlying structure of language and sometimes grouped together with grammaticality. In early stages, syntactic processes and speech processes remain preserved (Savundranayagam et al., 2005; Ferris and Farlow, 2013; Klimova et al., 2015). However, complexity of syntax in written language has been shown to be significantly associated with cognitive impairment (Aronsson et al., 2020). In addition, ML classification experiments have identified syntactic impairment in the AD Dementia groups (Fraser et al., 2016). Beyond identifying known language impairment, it is crucial to consider that speech and language processes do not occur in isolation and are intertwined with other cognitive and physical processes.

### Impaired Language vs. Impaired Speech

Impaired speech is the physical process of speaking involving the lungs, trachea, vocal chords and mouth whereas impaired language refers to deficits in the cognitive process of forming language with structure and meaning. While ML approaches are a powerful tool to estimate the utility of spontaneous speech features, interpreting them in a neuropsychological sense remains challenging. Although speech features are extracted from spoken language, this does not necessarily entail that they reflect language as a neurocognitive function as speech is confounded with multiple neurocognitive processes as well as gender, age and culture. As a result, not all well discriminating speech features can be assumed as evidence for the cognitive aspects of language deficits in AD dementia.

### Compound Cognitive Processes and the Picture Description Task

Cognitive, language, and speech processes are interdependent employing multiple aspects of cognition: retrieval from semantic and episodic memory, sustaining and dividing attention for error monitoring, as well as working memory for syntax production (Mueller et al., 2018). For instance, inability to recall a specific word—a semantic deficit—can result in a person with AD not being able to maintain concentration on the task—a pragmatic issue (Ferris and Farlow, 2013).

Since spontaneous descriptions of pictures are a compound cognitive performance of multiple neurocognitive functions and do not purely represent language impairment, when modeling impaired language processes embedded in speech, additional theoretical guidance and architecture within the ML experiments are needed to interpret speech-based features. It is not safe to assume that all well-discriminating ML features (in an AD vs. HC setup) are intuitively explainable, or even relevant, with respect to underlying cognitive processes. Spontaneous speech from the picture descriptions task is a compound of cognitive functions including language. Therefore, careful feature curation is needed to ensure that features are truly measuring language impairment and not just task performance.

### Natural Language Processing and the Picture Description Task

**Figure 1:**
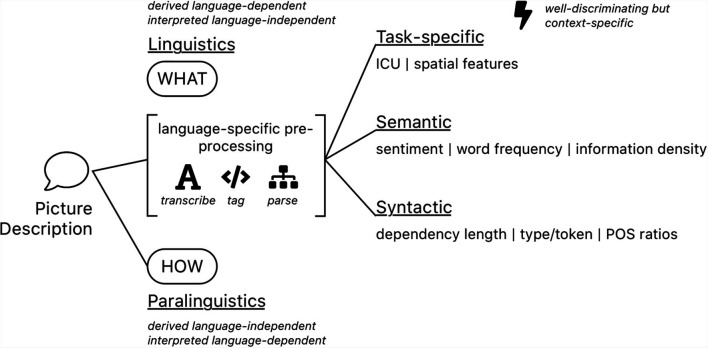
A schematic overview of feature kinds that are typically extracted from spontaneous speech picture descriptions. Some of them involve extensive pre-processing steps such as automatic speech recognition (ASR), part of speech tagging or sentence parsing and additional linguistic resources for calibration, others not.

Most qualitative analyses of spontaneous speech picture descriptions try to model cognitive impairment by leveraging a variety of computationally extracted features. Calz et al. (2021) reviewed 51 studies for dementia detection from the very common Cookie Theft Picture Description Task (CTP; Goodglass et al., 2001), collected and split 87 features into: rhythmic, acoustic, lexical, morpho-syntactic, and syntactic subgroups. Fraser et al. (2016) engineered features and categorized them into: part of speech, syntactic, grammatical constituency, psycholinguistics, vocabulary richness, information content, repetitiveness, and acoustic subgroups. Using factor analysis, they conclude on findings of semantic impairment, syntactic impairment, information impairment, and acoustic abnormality. For our analysis, we build off this finding to create four feature subsets: task-specific, semantic, syntactic and paralinguistic features (see also Figure 1). While it is arguably impossible to fully disambiguate each feature into a single category (Savundranayagam et al., 2005; Ferris and Farlow, 2013), we argue to evaluate features based on the following structure.

#### Task-Specific Features

In clinical practice, the CTP task is scored by counting the number of unique entities that a person mentions in the picture, referred to as information units (IUs). The individual counts of IUs in the CTP task (e.g., the number of times someone says cookie) are often used in automatic classification scenarios for cognitive impairment (Zraick et al., 2011; Fraser et al., 2016, 2019; Eyigoz et al., 2020). However, we argued that these individual counts are not indicative of semantically-motivated language impairment but rather represent task-specific performance or task completion. This is underpinned by the finding that most of the individual IU count features are not correlated with other classic psychometric language function assessments (Kavé and Goral, 2016). Fraser et al. (2016) found that including these features in ML experiment could be explained by information impairment as well as semantic impairment and represents a joint effort of multiple neurocognitive functions. In addition, IU count-based features are currently recognized as being task-specific also in state-of-the-art work on this topic (Robin et al., 2020). Thus, these features are treated as a measurement of general task performance in this study and not as indications of language impairment.

#### Semantic Features

It is generally accepted that one of the earliest characterizable impairments caused by AD dementia are semantic processes (Appell et al., 1982; Martin and Fedio, 1983; Bucks et al., 2000; Savundranayagam et al., 2005; Ferris and Farlow, 2013; Klimova et al., 2015). When modeling semantics, features are engineered to capture what is being said. In the CTP task, the semantics are constrained to what is happening in the image, allowing features to be extracted in an automatic and anticipated fashion. Here, semantic features are defined in the CTP as the high-level grouping of named IUs, commonly used by clinicians use to evaluate the task, and not the individual count of each IU. As an example, the number of times the patient says “girl” is not a generalizable representation of semantics but the total number of named IUs in the image can be used to measure ability to explore the semantic space. It has been shown that semantic measures, usually implemented in predefined IUs that represent the content of the to-describe picture, yield across the board good results in classifying between AD dementia and HC (for a review, see Mueller et al., 2018). Previous studies have reported that the AD group reports generic IU features (e.g., girl) without exploring more specific terms (e.g., sister, daughter; Eyigoz et al., 2020). We expect semantic impairment to be prevalent and evident between corpora and languages.

#### Syntactic Features

In this automatic scenario, syntactic features are engineered to represent the structure of language. This can manifest in a quantifiable way such as differences of sentence complexity or increased use of certain parts of speech. Other studies have reported significant AD dementia-related language impairments from picture descriptions as measured by syntactic features (Lyons et al., 1994; Kempler et al., 1998; Ahmed et al., 2013; Fraser et al., 2016; Yancheva and Rudzicz, 2016). This representation of language requires language specific resources in order to be calculated. We hypothesize these features to be moderately language dependent but some features to represent syntactic impairment that overlaps between languages.

#### Paralinguistic Features

Paralinguistic features—sometimes also referred to as acoustic, audio or speech features—are specifically appealing for automated speech analysis as they require minimal to no pre-processing and in theory capture the full variance of the acoustic signal and therefore the pathological speech behavior. The calculation of the features is often borrowed and repurposed from ASR systems, where the measures are done on the physical representation of the speech signal. There are multiple examples that successfully use paralinguistic features extracted from spontaneous speech picture descriptions to effectively discriminate between dementia and HC (Pakhomov et al., 2010; Satt et al., 2014; König et al., 2015; Fraser et al., 2016, 2019; Yancheva and Rudzicz, 2016). Due to the limited involvement of error-prone pre-processing steps (e.g., ASR to derive transcripts for further linguistic analysis) the use of paralinguistic features is often regarded as particularly robust and generalizable (Satt et al., 2014). In contrast, other studies found that paralinguistic features are particularly bad at modeling longitudinal trajectory of dementia or predict established clinical staging scores (Yancheva et al., 2015). From a theoretical point of view, we argue that paralinguistic features have great potential to model differences between AD dementia and HC within a certain data set but at the same time bear an equally great risk of over fitting to the particular language or data set. In terms of monitoring language impairment, it is very unlikely a clean proxy for language impairment in AD dementia can be obtained from speech features but at most for other cognitive (attention or executive functions), physical (lung capacity, vocal tract length) or pathological correlates (affective symptoms) associated with AD dementia (Alario et al., 2006; Baese-Berk and Goldrick, 2009; König et al., 2019).

## Materials and Methods

To investigate explainable and generalizable NLP approaches for automatically classifying between AD related language impairment and healthy controls, implemented the following three-step methodology:

1. First, a multilingual corpus of English and French spontaneous speech picture descriptions is introduced. Then, features are engineered and sorted into subgroups (task-specific, semantic, syntactic, paralinguistic) based on the aforementioned theoretical considerations. For each corpus, an identical set of features are extracted.
2. In a second step, taking advantage of the multilingual corpora, an inspection of cross-language correlations and statistical significance testing is done. Following the idea that well-differentiating features that model generalizable language impairment as a neurocognitive construct should be significant in both languages.
3. To arrive at explainable and generalizable classification results, ML experiments are conducted separately in the two different languages and in a multilingual setting. For each setting, a classification is done among all semantic, syntactic and paralinguistic features. This is compared to classification results where only “generalizable language” features are used. Generalizable language features are defined as semantic, syntactic and paralinguistics features that are significant in both languages.

By leveraging a multilingual approach, we aim to identify AD related language impairment that generalizes beyond a single corpus or language and models the processes of clinically observable language impairment.

### Participants

In this article we include 154 participants (78 healthy subjects) from two different languages (106 English speaking and 47 French speaking) drawn from two different available corpora (English, 2020 ADReSS INTERSPEECH challenge and French, EIT-Digital ELEMENT project); for a comprehensive overview of all demographics see Table 1.

**Table 1:** Sample characteristics for English and French samples.

Language	Diagnosis	N (M/F)	Age	Education	MMSE
English *N* = 106	HC	52 (23/29)	66.13 (6.52)	-	29.10 (1.00)
	AD	54 (24/30)	66.76 (6.61)	-	11.06 (5.49)
French *N* = 47	HC	25 (6/19)	75.40 (7.00)	12.80 (2.08)	28.56 (1.42)
	AD	22 (9/13)	81.59 (4.52)	10.91 (3.94)	18.36 (4.29)

*Age in years (SD), Education in years (SD) and score on MMSE cognitive screening with a max score of 30 (SD)*. Abbreviations: HC, Healthy Controls; AD, Alzheimer’s disease; MMSE, Mini Mental State Examination.

The English ADReSS sample (Luz et al., 2020) is a balanced (age- and gender-matched) subset of English DementiaBank (Macwhinney et al., 2011) of 53 HC and 54 confirmed AD patients. There are a total of 106 normalized recording and manually annotated transcripts of the cookie theft picture description task. This subset is derived from the DementiaBank corpus, which is part of the larger TalkBank project (Macwhinney et al., 2011). Patients were assessed between 1983 and 1988 as part of the Alzheimer Research Program at the University of Pittsburgh (for a detailed description of the cohort see Becker et al., 1994). Participants were referred directly from the Benedum Geriatric Center at the University of Pittsburgh Medical Center, and others were recruited through the Allegheny County Medical Society, local neurologists and psychiatrists, and public service messages on local media. Inclusion criteria were as follows: above 44 years of age, at least 7 years of education, no history of nervous system disorders or be taking neuroleptic medication, initial Mini-Mental State Exam (MMSE) score of 10 or greater and had to be able to give informed consent. Participants with dementia had a relative or caregiver acting as an informant. Participants received neuropsychological and physical assessment and were assigned to the “patient” group primarily based on a history of cognitive and functional decline, and the results of a mental status examination. In 1992—after the end of the study—the diagnosis of each patient was confirmed through clinical record and if available autopsy.

The French ELEMENT sample (König et al., 2018) contains 47 participants that completed the cookie theft picture description task. The initial participant pool was 179 subjects but only 47 participants were given the CPT task while the others were given a different spontaneous speech picture description and therefore are not considered in this study. Participants were recruited within the framework of a clinical study carried out for the EIT-Digital project ELEMENT, speech recordings were conducted at the Memory Clinic located at the Institut Claude Pompidou and the University Hospital in Nice, France. The Nice Ethics Committee approved the study. Each participant gave informed consent before the assessment. Speech recordings of participants were collected using an automated recording app which was installed on an iPad. The application was provided by researchers from the University of Toronto, Canada, and the company Winterlight Labs. Each participant underwent the standardized process in French Memory clinics. After an initial medical consultation with a geriatrician, neurologist or psychiatrist, a neuropsychological assessment was performed. Following this, participants were categorized into different groups: control participants (HC) that were diagnosed as cognitively healthy after the clinical consultation and patients that were diagnosed as suffering from Alzheimer’s disease and related disorders (AD). For the AD, the diagnosis was determined using the ICD-10 classification of mental and behavioral disorders (World Health Organization, 1992). Participants were excluded if they were not native speakers or had any major hearing or language problems, history of head trauma, loss of consciousness, addiction including alcoholism, psychotic or aberrant motor behavior or were prescribed medication influencing psychomotor skills. Among the 47 participants that performed the CPT, 22 participants were diagnosed with Alzheimer’s disease or related dementias (AD) and 25 participants with subjective memory complaints but no detectable dementia. A Kruskal–Wallis H test revealed significant age differences ($\chi_{\left(1\right)}^{2}$ = 9.79, *p* < 0.01) but no significant difference for education level.

### Spontaneous Speech Procedure

In both samples (DementiaBank subset and Dem@Care subset) participants completed a comprehensive protocol of assessments of which for this research only the recordings of the Cookie Theft Picture description task are relevant. In both samples, subjects provided informed consent to be recorded while describing the “Cookie Theft” picture from the Boston Diagnostic Aphasia Examination (Goodglass and Kaplan, 1983).

In this task, participants are shown a black and white image of a kitchen with multiple on-going antics while being instructed to “Tell me everything you see going on in this picture.” Testing personnel generally is not meant to provide any feedback during the descriptions of the participants. However, in some cases there is interaction recorded if for example the initial response of the patient is unreasonably brief, such as only a single sentence. Recordings had a mean duration of 62.63 s (*SD* = 35.83) sometimes including prompts from the examiner. The English corpus has an average duration of 70.92 s (*SD* = 36.92) and the French corpus has an average duration of 43.95 s (*SD* = 24.82). All recordings are transcribed according to CHAT protocol (Macwhinney, 1991).

### Feature Engineering

For each of the four categories defined previously (semantics, syntax, task-specific, and paralinguistic) features were engineered and then calculated using a program written in the Python programming language (Van Rossum and Drake, 2009; Version 3.7). The following section describes the computation of the features by sub-group. If a language-specific resource is used, the equivalent resource is used for each language in the data.

#### Task-Specific Features (*N* = 107)

Croisile et al. (1996) defined a set of general IUs that appear in the CTP task (e.g., girl, boy) and these IUs are mapped to a larger set of synonymous keywords (e.g., brother, girl). For instance, the boy in the picture may also be referred to as brother or son. This is done for the following IUs: boy, girl, woman, kitchen, exterior, cookie, jar, stool, sink, plate, dishcloth, water, window, cupboard, dish, curtain. A table of the mappings for each IU category to its keywords is provided in the Supplementary Materials for both French and English. For each IU, three features are computed: a binary value to see if the IU is mentioned, the count of times the IU is mentioned, and the ratio of the IU to all mentioned IUs. For spatial features, the CTP image is divided into different subgroups^1^. Three divisions of the image are considered: halves, quadrants and vertical stripes. Halves is where IUs are defined as being on the left side or right side. Quadrants breaks the image into four equal squares, north-east, north-west, south-east and south-west. Vertical stripes cut the image vertically into most-left, center-left, center-right and most-right (Goodglass and Kaplan, 1972). For each of the subsections the following features are calculated: word count, type-to-token ratio, keyword-to-word ratio, and percent uttered. For the division in halves, the number of switches between the sides is considered.

#### Semantic Features (*N* = 20)

Some semantic features utilize task specific resources, but model semantics by combining the defined IUs—and their mapped keywords—into refined, global semantic features rather than counting individual IUs. A table with the mappings between the IU and the keywords that make up the IU are provided in the Supplementary Materials for both English and French. Semantic features calculated with the IUs and keyword mappings are defined in Table 2. In addition to the features in the table, semantic features that do not rely on the IU definitions are also calculated. The Word Frequency package for python (Speer et al., 2018) is used to determine the mean, median, and max word frequency of all words as well as mentioned keywords. In addition, the mean, median and max word length is calculated for all words as well as the keywords. To gauge lexical richness of the responses, the type-to-token ratio (TTR) is calculated by dividing all unique words said by the total word count. The Moving-Average-Type-Token Ratio (MATTR) is calculated using a fixed window size of 10. For this measurement, a ratio of the number of distinct words in the sliding window is divided by the total count of words. For example, the TTR for words 1–10 is estimated followed by the TTR for words 2–11, then 3–12, and so on. The resulting TTRs are averaged, the estimated TTRs are averaged. Conceptually, the moving-average type–token ratio MATTR (Covington and Mcfall, 2010) calculates the TTR while reducing the influence that the length of the text has on the measure.

**Table 2:** Explanation of semantic features.

Example: There is a boy. The boy is a brother. He is stealing a cookie. The sister is watching.		
Feature name	Explanation	Example
**Number of Unique IU (num_unique_IU)**	The number of unique IU mentioned Higher means they mentioned more IU in the picture	3, boy and cookie, sister
**Number of Unique Keywords (num_unique_keywords)**	The number of unique keywords mention Higher means they either used more IU and/or used more lexical variety to describe the IU.	*4, boy, brother, sister and cookie*
**Number of Total keywords (num_total_keywords)**	Counts all mentions of the IU from the mapped keywords. Higher means they said more overall about the image.	*5, boy, boy, brother, cookie, sister*
**Unique IU Density (unique_IU_density)**	The number of unique IU (num_unique_IU) mentioned divided by the word count	num_unique_IU = 3; Word count = 18 3/18 = 0.1667
**Total IU Density (total_IU_density)**	Number of total IU (num_total_IU) divided by the word count.	num_total_IU = 5; Word count = 18 5/18 = 0.2778
**Keyword to non-keyword ratio** **(keyword_to_non_keyword_ratio)**	$\frac{num\;total\;keywords}{word\;count-num\;total\;keywords}$	num_total_keywords = 5; Word count = 18 5/(18–5) = 0.3846
**Unique IU efficiency** **(unique_IU_efficiency)**	The number of unique keywords (num_unique_keywords) divided by the word count.	num_unique_keywords = 4; Word count = 18 4/18 = 0.22
**percentage of IU mentioned** **(percentage_of_keywords_mentioned)**	The number of unique IU (num_unique_IU) mentioned divided by the total count of all IU words available in the image.	num_unique_IU = 3, all_IU_words = 16 3/16 = 0.1875
**Keyword Type Token Ratio (keyword_TTR)**	The number of unique keywords (num_unique_keywords) divided by the number of total IU (num_total_IU) mentioned.	num_unique_keywords = 4; num_total_IU = 5 4/5 = 0.8
**total IU efficiency** **(total_IU_efficiency)**	Number of total IU (num_total_IU) divided by the duration in seconds of the participant’s response.	num_total_IU = 5; duration = 15 s 5/15 = 0.33
**unique IU efficiency** **(unique_IU_efficiency)**	The number of unique IU (num_unique_IU) divided by the duration in seconds of the participant’s response.	num_unique_IU = 3; duration = 15 s 3/15 = 0.2

*Feature name contains the name of the feature in the text and the name of each feature use in images in parentheses. The explanation column explains how the feature is calculated. At the top of the table there is an example which is used in the example column to explain how each feature is calculated*.

#### Syntactic Features (*N* = 41)

To evaluate syntax, the mean words per sentence, word count and number of sentences are calculated. In addition, Spacy models are used to calculate the mean dependency length, median dependency length, max dependency length (Honnibal and Montani, 2017)^2^. Using Spacy language models, each participant’s response is part-of-speech tagged. The count of each tag, as well as the ratio of the POS tag count to total word count are computed. The following tags are considered: Adjective (ADJ), Adposition (ADP), Adverb (ADV), Auxiliary (AUX), Coordination Conjunction (CCONJ), Determiner (DET), Interjection (INTJ), Noun (NOUN), Numeral (NUM), Particle (PART), Pronoun (PRON), Proper Noun (PROPN), Punctuation (PUNCT), Subordinating Conjunction (SCONJ), Symbol (SYM), Verb (VERB, and Other (X). Specific ratios are calculated between nouns (NOUN) and verbs (VERB), pronouns (PRON) and nouns (NOUN), and determiners (DET) and nouns (NOUN). The open (ADJ, ADV, INTJ, NOUN, PROPN, VERB) to closed (ADP, AUX, CON, DET, NUM, PART, PRON) class ratio is also computed.

#### Paralinguistic Features (*N* = 208)

To extract paralinguistic features from the normalized wav files free, open-source python libraries, and praat (Boersma and Weenink, 2009) are used.

To characterize the temporal and content features of speech, the My Voice Analysis package^3^ is used. This package is developed by the Sab-AI lab in Japan to develop acoustic models of linguistics. This package interfaces the speech analysis research tool praat (Boersma and Weenink, 2009) with python, allowing the following features to be extracted from the wav recording: speech rate, syllable count, rate of articulation, speaking duration, total duration, pronunciation *posteriori* probability percentage score, and ratio of speaking to non-speaking. This package is also used to extract some prosodic features, specifically the mean, standard deviation, minimum, maximum, upper and lower quartile of the F0 value, or what is sometimes referred to as the pitch, in Hertz (Hz).

To represent the sound wave itself, features are borrowed from the ASR community using the Python Speech Features library. The original sound recording undergoes a series of transformations that yield a representation of the sound called the Mel Frequency Cepstrum (MFC). The MFC describes two crucial points of information from the voice to human anatomy; the first is the source (e.g., the lungs) and the second is the filter (e.g., place of articulation). The first transformation separates the source and filter from the signal and then maps this to the Mel scale which approximates the sensitivity of the human ear (Fraser et al., 2018). Typically, up to the first 14 coefficients are used as they represent the lower range frequencies of the vocal tract and yield most of the information (Hernández-Domínguez et al., 2018). This has been shown to be effective at identifying AD patients in previous literature (Dessouky et al., 2014; Rudzicz et al., 2014; Satt et al., 2014; Fraser et al., 2018; Panyavaraporn and Paramate, 2018; de la Fuente Garcia et al., 2020; Meghanani and Ramakrishnan, 2021). From this new representation, the first 14 coefficients of the MFC are extracted and the mean, variance, skewness and kurtosis are calculated for the energy (static coefficient), velocity (first differential), and acceleration (second differential). These are also calculated for the velocity and acceleration, where velocity is the difference between consecutive time steps, and acceleration is the difference between consecutive time steps for each velocity. Additionally, the mean, maximum, minimum and standard deviation of the root mean square value (RMS), centroid, bandwidth, flatness, zero crossing rate (ZCR), flatness, loudness, and flux of the spectrogram are calculated with the Librosa^4^ package.

### Inferential Statistical Analysis

After extracting identical feature sets from both corpora, features are evaluated with regard to their significance in differentiating between the two groups (AD and HC) using non-parametric group comparison and correlation analysis.

#### Significance Testing

For group comparisons, a non-parametric Kruskal–Wallis *H*-test for significance is done for each feature to test for significant group differences between the HC and AD samples. Due to the number of performed significance tests, we also report a Bonferroni adjusted probability. This is done separately for each language, meaning each feature has four significance values: English *p*-value, English adjusted *p*-value, French *p*-value, and French adjusted *p*-value. Significance was set at *p* < 0.05.

#### Correlation Analysis

Correlation analysis was used to arrive at a continuous numeric variable describing the ability of a feature in discriminating between AD and HC (AD/HC × feature value) which is at the same time comparable between both languages/samples; this is mainly relevant for plotting the discriminative power of feature in both languages and better visualizing the generalizability of the extracted features. For correlation values, a point-biserial correlation is calculated between each feature and the nominal group condition.

### Machine Learning Experiments

For all ML experiments, we investigate three classifiers: a classic logistic regression (LR) with an L2 regularization, a Support Vector Machine Classifier (SVM), and a simple neural approach with a multilayer Perceptron (MLP) using a logistic activation function and the regularization term (alpha) set to 0.01. All other parameters are left at their default setting. Due to the small size of the data sets in this article, we opted to maximize the available data using leave one out cross validation. For this method, one sample is held for testing and all other data points are used for training. This is repeated so that every sample in the data has been held out one time. While leave-pair-out cross validation is considered to be a less biased approach for binary classification because it exhaustively tries every possible combination, leave-one-out cross validation is a common training-testing split in this line of research (Cohen and Pakhomov, 2020; de la Fuente Garcia et al., 2020; Luz et al., 2020). Even on very small datasets, leave-pair-out cross validation is computationally expensive (Maleki et al., 2020). In order to keep our work comparable with prior and future studies, we opted to use leave one out cross validation as the best method for maximizing the available data while reducing training bias and maintaining reproducibility (Pahikkala et al., 2008; Fraser et al., 2019; Maleki et al., 2020).

Reported scores are the average across all iterations of the classification experiment. All ML experiments are implemented using the python library, scikit-learn^5^ (Pedregosa et al., 2011).

#### Selecting Generalizable Features

To determine which features capture language impairment that is not corpus-specific, the uncorrected Kruskal–Wallis significance testing described previously in statistical analysis (“Significance Testing” section) is used. Features are selected from each subgroup if they were found to be significant (*p* < 0.05) in both French and English and added to the “generalizable language” feature set. Task-specific features are excluded. The “generalizable language” features are listed in Table 3.

**Table 3:** Statistics as per feature set and language.

	Semantic features											
			English						French					
Feature	*r*_PB_	*m*_HC_	*m*_AD_	*χ*^2^	*p*	*p*_corr._	*r*_PB_	*m*_HC_	*m*_AD_	*χ*^2^	*p*	*p*_corr._
keyword_to_non_keyword_ratio	−0.43	0.16	0.11	19.3	***	***	−0.42	0.15	0.11	8.1	**	0.10
max_word_frequency_IU	−0.27	0.0003	0.0003	7.5	***	0.13	−0.34	0.0003	0.0002	5.3	*	0.47
mean_word_frequency_all	−0.37	0.0089	0.0072	14.7	***	***	−0.38	0.0075	0.0066	6.8	**	0.20
**num_unique_IU**	−0.60	10.94	6.87	37.8	***	***	−0.64	10.24	5.50	18.6	***	***
**num_unique_keywords**	−0.60	11.83	7.26	37.3	***	***	−0.60	11.40	5.73	16.6	***	**
**percentage_of_keywords_mentioned**	−0.60	0.10	0.06	37.3	***	***	−0.60	0.07	0.04	16.6	***	**
total_IU_density	−0.42	0.14	0.10	18.7	***	***	−0.36	0.14	0.11	5.9	**	0.34
**total_IU_efficiency**	−0.54	0.26	0.16	30.2	***	***	−0.46	0.34	0.21	9.8	**	*
**num_total_keywords**	−0.43	15.42	10.46	19.3	***	***	−0.57	13.44	6.41	15.1	***	**
unique_IU efficiency	−0.56	0.18	0.10	32.4	***	***	−0.40	0.27	0.17	7.2	**	0.16
unique_IU ratio	−0.45	0.10	0.07	21.2	***	***	−0.34	0.11	0.09	5.5	*	0.43
	**Syntactic features**											
	**English**						**French**					
**Feature**	***r*_PB_**	***m*_HC_**	***m*_AD_**	***χ*^2^**	***p***	***p*_corr._**	***r*_PB_**	***m*_HC_**	***m*_AD_**	***χ*^2^**	***p***	***p*_corr._**
ADP_count	−0.28	7.83	5.39	8.0	**	0.20	−0.50	14.44	6.95	11.4	***	*
**ADP_ratio**	−0.34	0.06	0.05	12.3	***	*	−0.51	0.13	0.09	11.9	***	*
AUX_ratio	−0.35	0.10	0.09	12.7	***	*	0.30	0.04	0.06	4.1	*	1.00
DET_count	−0.26	17.35	13.52	7.3	**	0.31	−0.45	17.72	9.95	9.4	**	0.09
DET_ratio	−0.43	0.15	0.12	19.3	***	***	−0.32	0.17	0.14	4.7	*	1.00
**NOUN_count**	−0.34	21.12	15.59	11.9	***	*	−0.49	20.76	11.09	11.0	***	*
NOUN_ratio	−0.48	0.18	0.14	23.8	***	***	−0.38	0.19	0.15	6.7	**	0.42
PRON_ratio	0.25	0.07	0.09	6.4	*	0.51	0.51	0.11	0.18	12.1	***	*
PUNCT_count	0.21	15.15	20.69	4.7	*	1.00	−0.38	1.06	0.32	6.5	*	0.47
PUNCT_ratio	0.36	0.13	0.18	13.8	***	**	−0.34	0.01	0.00	5.4	*	0.91
	**Paralinguistic features**											
	**English**						**French**					
**Feature**	***r*_PB_**	***m*_HC_**	***m*_AD_**	***χ*^2^**	***p***	***p*_corr._**	***r*_PB_**	***m*_HC_**	***m*_AD_**	***χ*^2^**	***p***	***p*_corr._**
bandwidth_mean	0.22	2,022.85	2,153.46	5.0	*	1.00	0.32	2,176.25	2,323.75	4.8	*	1.00
energy_skewness	0.23	0.17	0.32	5.4	*	1.00	0.47	-0.26	0.49	10.4	**	0.26
mfcc1_mean	−0.20	-1.87	-4.43	4.4	*	1.00	−0.36	-5.14	-7.87	6.0	*	1.00
mfcc1_skewness	0.23	0.19	0.48	5.8	*	1.00	0.44	-0.31	0.12	8.8	**	0.63
mfcc10_kurtosis	0.23	0.73	1.00	5.5	*	1.00	0.37	0.40	0.67	6.4	*	1.00
mfcc4_kurtosis	0.22	0.92	1.47	4.9	*	1.00	0.41	0.51	1.02	7.7	**	1.00
normalized_loudness_std	−0.34	0.20	0.18	11.8	***	0.12	−0.56	0.23	0.20	14.4	***	*
ratio_speaking	−0.27	0.46	0.37	7.6	**	1.00	−0.57	0.64	0.48	15.2	***	*
speech_rate	−0.28	1.92	1.41	8.5	**	0.73	−0.47	3.12	2.32	10.2	***	0.28

Point-biserial correlation coefficient *r*_PB_, correlating each feature with the nominal group variable (AD, HC), feature means for HC and AD, *χ*^2^ value of the non-parametric Kruskal–Wallis *H*-test for group differences between AD and HC, *p*-value and Bonferroni-corrected *p*-value. Significances: *** 0.001, ** 0.01, * 0.05. Feature names in Bold indicate that they are significant after Bonferroni-correction in both languages.

#### Experiment Scenarios

Thus far, we have presented two datasets, French and English (“Participants” section, Table 1). By concatenating these two datasets, we generate a third multilingual dataset. In addition, two feature groupings have been proposed; Language features defined as all features in the semantic, syntactic and paralinguistic features [for reference see “Semantic Features (*N* = 20),” “Syntactic Features (*N* = 41)” and “Paralinguistic Features (*N* = 208)” sections, and Figure 1] and a subset of these features that are considered to be the generalizable language feature set (“Selecting Generalizable Features” section).

To investigate the performance of the generalizable language feature set, six experimental scenarios are conducted in a binary classification scenario (HC vs. AD). For the first three experiments, English, French and multilingual models are trained using all language features. For the next three experiments, English, French and multilingual models are trained using the generalizable language features. We then compare the performance of the language feature set and the generalizable language feature set to see if the generalizable features help or hurt classification performance.

#### Establishing a Baseline

To relate these experiments to previous work, we train a baseline model that uses all feature subgroups (semantic, syntactic, task-specific and paralinguistic) in a classification with the previously described English dataset. This situates our methods and results in comparison to the recent ADReSS challenge at Interspeech 2020. The goal of this challenge was to use spontaneous speech picture descriptions to differentiate between AD and HC.

In addition to the experimental scenarios and baseline, we create a baseline classification experiment using only age to consider the affects that the unmatched French population has on the multilingual ML experiment.

#### Evaluation

For classification performance, Area Under the Receiver Operator Curve (AUC) is reported for each experiment scenario described in “Experiment Scenarios” section. Confusion matrices (Bateman et al., 2012; König et al., 2018) are reported for the multilingual model with the generalizable language feature set. A matrix is reported for the overall classification and then the error is broken down by individual language to investigate if the multilingually trained classifier performs equally in both languages.

## Results

Results are reported from the two methodological scenarios: inferential statistical analysis and ML experiments.

### Inferential Statistical Analysis

**Figure 2:**
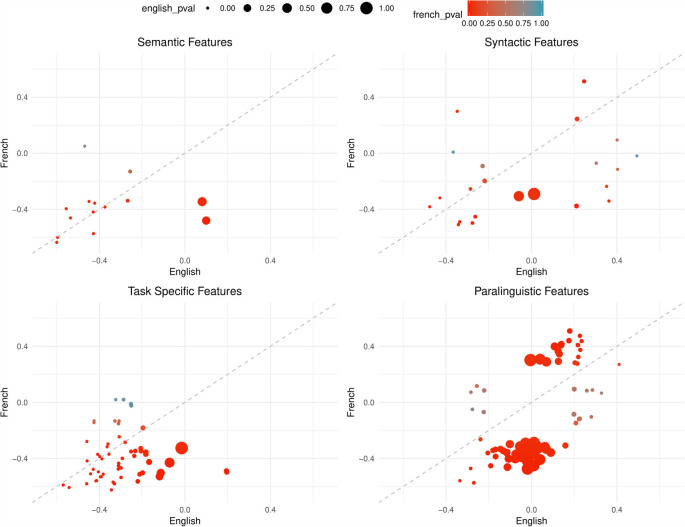
Points are plotted by correlation values (point-biserial correlation coefficient *r*_PB_, correlating the feature with the group AD vs. HC) with French on the Y-axis and English on the X-axis for each feature subgroup. The significance value (as by Kruskal–Wallis non-corrected significance test *p* < 0.05) is visualized by point color for French and point size for English. Points closer to the dashed line perform equally well in both languages. This figure contains all features that are significant in EITHER French or English, not necessarily both.

**Figure 3:**
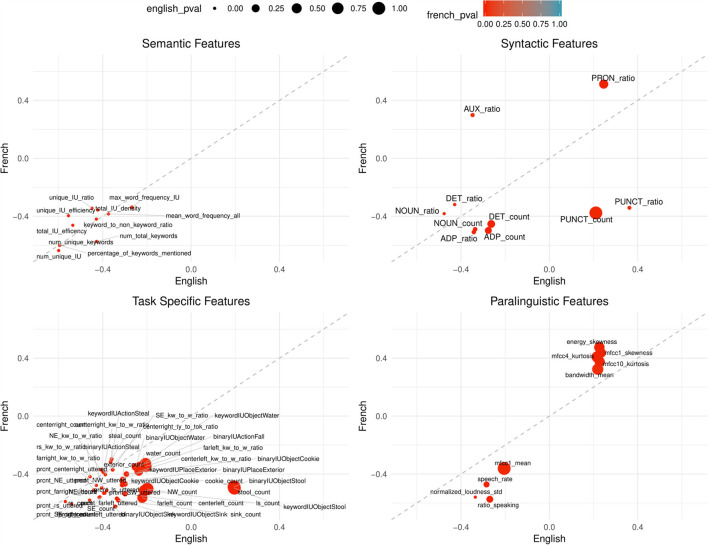
Points are plotted by correlation values (point-biserial correlation coefficient *r*_PB_, correlating the feature with the group AD vs. HC) with French on the Y-axis and English on the X-axis for each feature subgroup. The significance value (as by Kruskal–Wallis non-corrected significance test *p* < 0.05) is visualized by point color for French and point size for English. Points closer to the dashed line perform equally well in both languages. This figure contains all features that are significant in BOTH French AND English. Feature labels are added to each point.

Comparing the overall correlation and significance trends in Figures 2, 3, semantic and task-specific features display similar patterns. In general, these features are negatively correlated in both French and English where AD has lower averages than healthy controls. For syntactic and paralinguistic features, both negative and positive correlations are observed. Paralinguistic features show the most language-specific behaviors, where a mild language preference can also be seen in syntactic features, indicated by points that are far from the dashed line.

Following our above-introduced feature categories, we evaluated statistical significance in differentiating between both groups, AD and HC. Of all features calculated, 30% of task-specific, 28% semantic, 39% syntactic features and 65% of paralinguistics features are not significant in either French or English before significance correction. Before correction, 43% of task-specific, 52% of semantic, 24% of syntactic, and 4% of paralinguistic features of the initially extracted features are significant in both French and English (see also Table 3). However, due to the large amount features tested (*N*_total_ = 377), after Bonferroni correction only a fraction of the features remain significant in both languages; 9% task-specific, 24% of semantic, 5% syntactic, and 0% paralinguistic.

#### Task-Specific Features

Among 107 calculated task-specific features, 32 features are not significant in either French or English, roughly 30%. With significance correction, 75 features are significant in either French or English; 46 features in Both, 20 features in French-only, and nine features in English-only. After significance correction, 10 features remain significant for both languages, approximately 9% of all task-specific features.

#### Semantic Features

Among 21 calculated features, 15 features are significant in either French or English; two features in French-only, two features in English-only and 11 features in both. While the semantic subgroup has the least calculated features, it has the highest percentage of significant features (approximately 24%) after Bonferroni correction: number of unique IU, number of unique keywords, percentage of keywords mentioned, total IU efficiency, and number of total keywords. For all the significant features in English and French, the AD condition shows lower averages in comparison to the control group (HC).

#### Syntactic Features

In either language, 25 of the 41 syntactic features are significant in either French or English; 10 features in both, two features in French-only, and 13 features in English-only. After significance correction, noun count and adposition ratio are significant in both languages. For both features, the AD group shows lower averages than healthy controls.

#### Paralinguistic Features

In either French or English, 72 features among 208 calculated paralinguistic features are significant: nine features in both, 45 features in French-only, 18 features in English-only After significance correction, no features are significant in English and two features are significant in French; ratio of speaking to the full sample duration and the standard deviation of normalized loudness. In both cases, the AD group shows lower averages in comparison to the control averages.

In the paralinguistic subplot of Figure 2, features are highly polarized as shown by the clustering of points on either side of the dashed line, indicating very little feature overlap between the languages with weaker correlations—especially for English—in comparison to the other feature subsets. By looking at Figure 3 where features are significant in English and French, lower correlations and the features that are highly polarizes towards one language do not appear in the sub-graph. Among 208 features, only nine features are significant, before correction, and the remaining features are more highly correlated with French than English.

### Machine Learning Experiments

**Figure 4:**
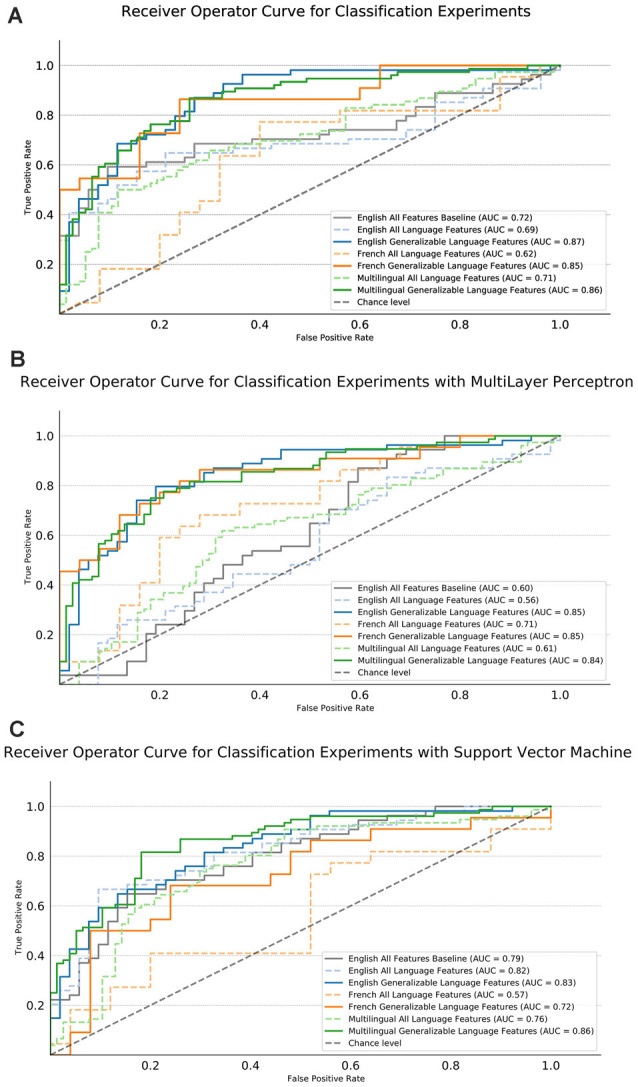
Area Under Curve (AUC) performance results of the machine learning (ML) experiments. English and French for the respective samples separately, multilingual is for the joint classification, multilingual significance testing for feature selection (Generalizable) or using all features (ALL) and using only semantic, syntactic, and paralinguistic features (Language Features). The gray dashed line indicates chance performance of the models. The English (blue), French (orange), and Both (green) models trained with semantic, syntactic, and paralinguistic features are shown with a dashed line. The English, French and Multilingual models trained with the significant, generalizable features in English and French are indicated by the solid lines in the same color, respectively.

Machine learning model performances are visualized for the baseline and experimental scenarios in Figure 4. Not included in the graph is the addition classifier for age. All the multilingual experiments were below chance (AUC = 0.5) for age: LR had an AUC of 0.49, SVM had an AUC of 0.38, and the MLP had an AUC of 0.40. This leads us to believe that age is not a good distinguisher between the HC and AD groups for the generalizable experiments. However, it does not eliminate age as a factor from this research and future experiments studies should replicate these findings with age, gender and education balanced data sets to control for possible external conflicting factors.

For the experiments with the LR, all of the models trained with generalizable language features outperform both their respective All language feature models and the English all features baseline. The baseline scenario, the English model with all features, is shown in a solid gray and performs with an AUC of 0.7. English gains 18 points, French gains 23 points, multilingual gains 14 points of AUC over their ALL models. The highest AUC score is nearly tied for the English selected with an AUC of 0.87 and both selected model with an AUC of 0.86. The French select is close in performance with an AUC of 0.85.

For experiments using the SVM, the multilingual generalizable model out-performs all other models reaching an AUC of 0.86. This model improves 10% over the all language feature model. In both English and French, the single language generalizable models outperform their respective all language feature models. This is more so the case for French where there is an improvement of 15 points, whereas English only improves by 1 point.

For the experiments using the MLP, we see a minor performance drop between the model with ALL baseline features and only generalizable language features. However, in both English and French we see a large performance increase when using only the generalizable features, with both the French and English models reaching an AUC of 0.85. For the multilingual MLP model, we see a mirrored pattern with the LR but slightly lower performance. Overall, we see a 23-point AUC increase when using the generalizable language features in the multilingual scenario, yielding a 0.84 AUC.

We see a similar pattern of error for both the LR, SVM, and MLP models. For the multilingual LR model trained with generalizable language features, the overall error rate is 22.22% and English (22.64%) and French (21.28%) exhibit roughly the same level of error. For the SVM, a slightly lower error of 20.26% is found with a similar split of error between English (20.75%) and French (19.15%). The same result is achieved using the MLP, with a 21.56% overall error and a slightly high, although still comparable, level of the error in both languages (20.75%) English and (23.40%) French.

For both model types, the number of false positives—the case of classifying a control as AD—by language in the multilingual select model make up roughly 30% for French error (27% for the MLP) and 54% for English (59% for the MLP) error. In both models, the English samples have a balanced split of error, but the French model suffers from elevated false positive error. However, this is not the case for the SVM where French (43%) and English (47%) are more balanced in their false positive rate.

## Discussion

This article addresses the research gap between the clinical understanding of language impairment (as a neurocognitive functions impairment) apparent in everyday spontaneous speech and recent NLP techniques used together with ML for speech-based classification of AD against healthy control subjects. We propose to: (1) gain insights into AD-related language impairment and its cognitive sub-processes through multilingual NLP feature statistics (generalizing beyond one single language as a cultural phenomenon); and (2) train robust ML models capturing cognitive language impairment in AD with these generalizable features and compare to other methods on the same dataset.

### Generalizable NLP Features of Language Impairment in AD

#### Semantic Features

While the semantic subgroup consists of the lowest number of features, it has the largest number of significant features after Bonferroni correction: number of unique IU, number of unique keywords, percentage of keywords mentioned, total IU efficiency, and number of total keywords. For all the significant features in English and French, the AD condition shows lower averages in comparison to the control group (HC).

Lower averages in number of unique IU, number of unique keywords, number of total keywords, and percentage of keywords mentioned indicates reduced lexical variety and exploration of the available semantic space by the AD group, which is indicative of impaired semantic processes. In addition, there is a reduced semantic efficiency where the AD group is exploring fewer IUs in the same amount of time as controls. For AD patients we found lower overall information efficiency of the uttered descriptions (e.g., total IU density) as well as lower lexical variety with which AD patients described and referred to different IUs in the picture (ratio of unique keywords to all keywords mentioned) in both languages. This is in line with earlier work that finds a decreased semantic efficiency (semantically empty speech) in AD patients’ spontaneous speech from picture descriptions (Ahmed et al., 2013; Fraser et al., 2016) but also from other language production tasks (Snowdon et al., 1996; Le et al., 2011).

Overall, semantic features indicate generalizable semantic impairment in AD. Of the feature subgroups, semantic featuresgeneralize the best between the languages, supporting the argument that these features or not task-specific but measure more general semantic abilities. The AD populations, regardless of language, show deficits in semantic scores compare to the health control group.

#### Syntactic Features

Syntactic features show generally weaker correlations with the pathological state (AD vs. HC) than semantic features. In comparison to the semantic features, syntactic features display a trend of mild language specific behavior (compare also the distance from the dashed line as well as the color/French or size/English of points in Figure 2). An interesting finding is the opposing correlation trends for punctuation count and ratio (positive for English and negative for French) and auxiliary ratio (negative for English, positive for French) continuing to indicate that there are syntactic features that are not generalizable for clinical populations because of language. Previous work has shown deficits in determiners, auxiliaries and reduced grammatical structure (Eyigoz et al., 2020). However, the remaining significant syntactic features after correction are adposition ratio and noun count. On average, the AD dementia group use less nouns and adpositions^6^.

Adpositions, specifically prepositions, are words used before a noun or pronoun to show time or spatial relationships. For example, in the sentence *the boy is reaching into the cookie jar*, *into* is a preposition showing the spatial relationship of *the boy* to *the cookie jar*. Preposition deficits for AD have been found in Brazilian Portuguese (Alegria et al., 2013). Another study—arguing that spontaneous speech mirroring the decline of effective spatial reasoning in language production—found that AD and HC used the same number of locative/stative prepositions (e.g., in, on, and at) but found significant differences for directional/dynamic prepositions (e.g., into, onto, from, and to; Bosse, 2019).

Although pronoun ratio is only significant in French after the correction, combining this finding with the significant difference in noun count could produce interesting deductions. Between the groups, AD dementia group has a lower average noun count but a greater average pronoun count. Grossman et al. (2007) used new verb acquisition to show that, in comparison to controls, AD dementia patients had fragmented knowledge acquisition. The AD group was able to grammatically use the verb but did not retain its semantic meaning. This could lend insights into the increased pronoun ratio and decrease noun count, where the AD group is not able to recall the semantic names of the IUs in the picture (e.g., boy, brother) and compensates using pronouns (e.g., he). This may be directly related to semantic AD-related language impairment (as described above), where a person uses ambiguous terms (pronouns) instead of specific lexicals (nouns; Savundranayagam et al., 2005; Ferris and Farlow, 2013; Klimova et al., 2015).

While some studies report reduced syntactic complexity in AD patients in earlier detection ML scenarios for the CTP (Fraser et al., 2016), others show contrary findings showing no association between syntactic complexity and cognitive pathology at early stages (Mueller et al., 2018). Evidence from other cognitive tasks show impaired syntax early in disease progression from free spontaneous speech as elicited by questions (Croisile et al., 1996) or written picture descriptions (Kemper et al., 2001).

These findings lead us to believe that syntactic impairment is present but could be confounded as compensation for the profound semantic deficits or other cognitive processes in AD dementia related language impairment.

#### Paralinguistic Features

For the group of paralinguistic features only around 10% of the initially extracted features were kept after multilingual significance check. Although paralinguistic features are typically reported as important well-classifying features in almost all AD language investigations using computer-aided automatic speech analysis in combination with ML (Pakhomov et al., 2010; Satt et al., 2014; König et al., 2015; Fraser et al., 2016, 2019; Yancheva and Rudzicz, 2016) and explicitly mentioned as robust solutions to the problem (Satt et al., 2014), we find the contrary: the majority of state-of-the-art paralinguistic features do not generalize between languages and therefore are probably not modeling language impairment in AD as a neurocognitive function. Therefore, we argue that they need further clinical investigation to be used as an argument about language impairment in AD.

On the other hand, the question remains why paralinguistic features model differences between healthy and pathological spontaneous speech so well in ML classification scenarios. It could be that they represent variance from other factors such as affective correlates like apathy, which has been shown to affect paralinguistic properties of speech and is a common comorbidity in AD (König et al., 2019), or other non-language neurocognitive functions such as executive functions. For example, we found a lower speech rate in AD patients in both languages which can be interpreted as evidence for a generally impaired psychomotor speed which is highly related with additional factors such as age and executive functions (Keys and White, 2000). However, it is also very likely that from the large amount of extracted paralinguistic features, the “significant” ones just represent statistical artifacts. This can be argued as after Bonferroni correction none of the paralinguistic features yields significance in both languages. This result illustrates well the paradox of paralinguistic features that are highly discriminative in AD vs. healthy control ML experiments but according to traditional interference statistics standards would be considered an artifact suffering from alpha error accumulation. Even without the multilingual generalizability consideration, this methodological paradox typically is disregard in state-of-the-art research combining NLP features for AD classification with ML.

After correction, no features were significant in both languages for English and only two features were significant in French: the ratio of speaking to the full sample duration and the standard deviation of normalized loudness. In both cases, the AD dementia group shows lower average scores than controls. The AD group speaks less overall which can be interpreted as a proxy for overall amount of language production in this task. This possibly reflects semantic, but also multiple other cognitive processes, as previously stated. A lower standard deviation of normalized loudness for the AD group indicates less change in speaking volume as compared to the control group. This could be indicative of common AD-related affective comorbidities such as apathy (König et al., 2019) which result in a less expression and variation in speech patterns.

#### Overall Findings

Overall, our investigation of generalizable NLP features for language impairment in AD robustly confirms AD patients’ semantic impairment in terms of low information efficiency and therefore semantically empty language. This cardinal semantic syndrome can be also additionally confirmed by increased syntactic compensation (using ambiguous terms instead of precise lexical-semantic terminology). Beyond this, we find reduced usage of prepositions, independent of the language, which could be indicative of the earlier-reported decreased complexity in AD language production but more research needs to be done to determine if this is syntactic impairment or confounded by other cognitive processes. Finally, we found almost no paralinguistic features that are indicative of a robust global hence cognitive language impairment in AD except for those who proxy either semantic deficits or affective comorbidities—the latter one indicating a non-causal correlation rather than a robust signal on language impairment in AD.

### Machine Learning Models With Generalizable and Explainable Features

#### Comparison to Baseline

The English baseline classifier with all features (on the same data set as Cummins et al., 2020; Farrús and Codina-Filbà, 2020) achieved an AUC of 0.72 and accuracy of 69.7% using a LR classifier. In comparison, the English classifier with generalizable language features achieved an AUC of 0.87 and an accuracy of 76.4% using a LR model.

On the balanced DementiaBank dataset using both linguistic and paralinguistic features, an 87.5% classification accuracy was achieved using a Random Forest classifier (Farrús and Codina-Filbà, 2020) and an 85.2% using a fusion deep learning approach (Cummins et al., 2020). On a different subset of 167 samples from DementiaBank, combining linguistic and paralinguistic features yielded an 81% accuracy (Fraser et al., 2016).

For multilingual approaches, only semantic word embeddings based on IU features were used to classify in a Swedish and English early detection setting with an 72% accuracy in Swedish and 63% accuracy in English (Fraser et al., 2018). French and English were used to train IU-level language models. The authors report a 0.89 AUC between AD and HC, the best model being trained on both languages (Fraser et al., 2019). The authors could not find any studies where a multilingual approach combined linguistic and paralinguistic features.

Other approaches, not explicitly extracting features, have been used for high performance classifiers on other subsets of the DementiaBank data. Namely, modeling the language of each population and then using perplexity scores (Fraser et al., 2018; Cohen and Pakhomov, 2020) has shown promising results producing interpretable models and reporting AUC scores of 0.93 (Cohen and Pakhomov, 2020). For a more in-depth overview of other methods used for automatic classification used for DementiaBank, please see de la Fuente Garcia et al. (2020).

#### Model Discussions

Looking at the ML experiments, the multilingual method of feature selection to identify generalizable language features drastically improved every ML performance.

For English, between the baseline with all features and using only language features, there is a small dip in performance when the task-specific features are removed. However, the best English, French and multilingual model performances is with the generalizable language features. More importantly, the performance increase is not only in the multilingual classifier, but a similar level of error is maintained between both languages separately (see Table 4). This finding is backed up by the confusion matrices that show a similar distribution of error types across the board. In both languages, as well as in the overall classifier, a comparable number of AD patients were wrongly classified as healthy (false negatives) and a comparable number of healthy subjects got wrongly classified as AD patients (false positives).

**Table 4:** Confusion matrices for the final robust classifier without task-specific features using multilingual significance feature selection.

LR Results			
**English and French without task-specific features and feature selection (Error Rate = 22.22%)**			
		Ground Truth (Diagnosis)	
		**True**	**False**
Classification Prediction	AD (positive)	58 (AD/AD)	16 (AD/HC)
	HC (negative)	61 (HC/HC)	18 (HC/AD)
**English classifications from the above joint ML scenario (Error Rate = 22.64%)**			
		Match to Ground Truth (Diagnosis)	
		**True**	**False**
Classification Prediction	AD (Positive)	43 (AD/AD)	13 (AD/HC)
	HC (Negative)	39 (HC/HC)	11 (HC/AD)
**French classifications from the above joint ML scenario (Error Rate = 21.28%)**			
		Match to Ground Truth (Diagnosis)	
		True	False
Classification Prediction	AD (Positive)	15 (AD/AD)	3 (AD/HC)
	HC (Negative)	22 (HC/HC)	7 (HC/AD)
**SVM Results**			
**English and French without task-specific features and feature selection (Error Rate = 20.26%)**			
		Ground Truth (Diagnosis)	
		True	False
Classification Prediction	AD (Positive)	59 (AD/AD)	14 (AD/HC)
	HC (Negative)	63 (HC/HC)	17 (HC/AD)
**English classifications from the above joint ML scenario (Error Rate = 20.75%)**			
		Match to Ground Truth (Diagnosis)	
		True	False

Classification Prediction	AD (Positive)	42 (AD/AD)	10 (AD/HC)
	HC (Negative)	42 (HC/HC)	12 (HC/AD)
**French classifications from the above joint ML scenario (Error Rate = 19.15%)**			
		Match to Ground Truth (Diagnosis)	
		True	False
Classification Prediction	AD (Positive)	17 (AD/AD)	4 (AD/HC)
	HC (Negative)	21 (HC/HC)	5 (HC/AD)
MLP Results			
**English and French without task-specific features and feature selection (Error Rate = 21.56%)**			
		Ground Truth (Diagnosis)	
		True	False
Classification Prediction	AD (Positive)	59 (AD/AD)	16 (AD/HC)
	HC (Negative)	61 (HC/HC)	17 (HC/AD)
**English classifications from the above joint ML scenario (Error Rate = 20.75%)**			
		Match to Ground Truth (Diagnosis)	
		True	False
Classification Prediction	AD (Positive)	41 (AD/AD)	13 (AD/HC)
	HC (Negative)	39 (HC/HC)	13 (HC/AD)
**French classifications from the above joint ML scenario (Error Rate = 23.40%)**			
		Match to Ground Truth (Diagnosis)	
		True	False
Classification Prediction	AD (Positive)	14 (AD/AD)	3 (AD/HC)
	HC (Negative)	22 (HC/HC)	8 (HC/AD)

*The first matrix shows the overall classification result of the model trained on the multilingual data. To ensure this model is not favoring one language, results are further broken down by language in the following matrices. Error is indicated by the false column where a false positive (AD/HC) is the case where a healthy control is classified as having AD and the False negative (HC/AD) is classifying a person with AD as a healthy control. The error rate is reported as all falsely classified participants divided by all participants*.

It has been shown early on that ML classification of AD and healthy subjects can benefit from a transfer learning approach between multiple languages (Fraser et al., 2019). However, we can show that in spontaneous speech picture descriptions a theory driven and generalizable approach to underlying features not only show good classification results between AD and healthy subjects but at the same time provides clinically-supported evidence of language impairment from spontaneous speech in AD.

Therefore, we conclude that there is evidence of language impairment in AD in everyday spontaneous speech and that this impairment could be driven by a language impairment in the neurocognitive sense. Evidence for this claim is provided by language-independent language impairments as robustly measured by linguistic (semantic and syntactic) and marginally also paralinguistic properties. This is in line with previous research on AD language impairments from traditional clinical research (Kempler, 1995; Taler and Phillips, 2008; Szatloczki et al., 2015).

## Conclusion

This study set out to investigate the robust, generalizable detection of language impairment from spontaneous speech in AD dementia through multilingual ML, with the goal of generating insights between both clinical and NLP researchers.

Based on the proposed methodology, we show possible language impairment in AD in a neurocognitive sense of language that is observable in everyday spontaneous speech. Our approach shows that task-independent language features of AD deteriorated speech point towards neurocognitive language impairments. The primary insights are situated in current clinical understanding of AD dementia related language impairments; There is a theorized primary semantic deterioration but also evidence of a milder syntactic impairment that is confounded by multiple other cognitive processes. In addition, the results support that language impairment could be measured by clinically-motivated NLP techniques without sacrificing overall performance.

The adjacent multilingual feature inspection shows that the feature categories correlate differently between both languages with regard to the significance of their features. This observation is of relevance for the research community interested in detecting language impairment in AD from spontaneous speech picture descriptions because language as a neurocognitive symptom has been found to be impaired in AD for different languages (Ahmed et al., 2013; Szatloczki et al., 2015; Mueller et al., 2018) even though AD itself is heterogeneous in the way it effects individuals (Lam et al., 2013; Ferreira et al., 2018). Hence, we highlight that by catering for explainability and generalizability by design of the ML experiments, research can not only generate efficient clinical applications of NLP methods for AD detection from spontaneous speech but also result in clinically actionable insights.

## Limitations and Future Work

The authors would like to acknowledge two main limitations in this study. First, A small clinical data set comes with many challenges. Ideally, to evaluate the ML models, we would use both a training dataset and held-out test set. Unfortunately, this is not available for the French data. Due to the lack of a held-out test set, ML scores could be artificially inflated.

Second, it is possible that poor performance by the paralinguistic features could be confounded by multiple factors: such as gender, the significant difference in age for the French population, and the audio quality of the recordings in DementiaBank. Age and gender have been shown to influence speech patterns and pitch range due to anatomical differences. Future work should investigate what impact these factors has on the explainability and generalizability of paralinguistic features. To support the results in this article, future work should try to replicate this study with more data as well as populations matched by age, gender, and education.

To validate the results presented in this study, future work should investigate this methodology on other clinical tasks that produce spontaneous speech to see if finds hold in more scenarios.

While we used ML to demonstrate that application of generalizable language features, we did not try any optimization techniques to boost results. Future work could look at other classifiers or tuning techniques to improve classification results.

## Data Availability Statement

The data analyzed in this study is subject to the following licenses/restrictions: access to demcare must be granted by the principle investigators; Alexandra König can be reached *via* alexandra.konig@inria.fr. Requests to access these datasets should be directed to Alexandra König, alexandra.konig@inria.fr.

## Author Contributions

JT and HL contributed equally to this article. AK led the French data collection as well as contributed to the clinical interpretation of the presented approach. All authors contributed to the article and approved the submitted version.

## Conflict of Interest

JT and AK own stock in the digtial speech biomarker company ki elements UG (haftungsbeschränkt). The remaining author declares that the research was conducted in the absence of any commercial or financial relationships that could be construed as a potential conflict of interest.

## Footnotes

^1^Implementation based on https://github.com/vmasrani/dementia_classifier

^2^https://universaldependencies.org/u/pos/

^3^https://pypi.org/project/my-voice-analysis/

^4^https://github.com/librosa/librosa

^5^scikit-learn version 0.23.2

^6^A combination of prepositions and postpositions. Postpositions in French seldom occur in spoken language and the only accepted postposition in English is *ago*. Therefore, adpositions, in this application, are assumed to be prepositions.
