# Supplementary material for: Language Impairment in Alzheimer’s Disease—Robust and Explainable Evidence for AD-Related Deterioration of Spontaneous Speech Through Multilingual Machine Learning
Source: Front Aging Neurosci. 2021 May 19;13:642033. doi: 10.3389/fnagi.2021.642033 (PMC8170097; doi:10.3389/fnagi.2021.642033)
Supplement: Supplementary file 1 [file Table_5.pdf]

Table 5. A table containing the scoring scheme used for the cookie theft picture in English and French. Each information unit has a set of mapped keywords in French and English.

Information Unit (IU)	French (Keyword)	English (Keyword)
**boy**	Garçon,fils, fiston, frère, enfant, enfants	Boy, son, child, kid, school boy
**cookie**	cookie, cookies, biscuits, gâteau, gâteaux, biscuit, tarte, pâtisserie, régal, bonbon, bonbons, sucré'	Cookie, cookies, biscuits, cake, biscuit, tart, pastry, treat, sweet, sweets, sugar
**cupboard**	placard, coffre, étagère, tablette, meuble	cupboard, cabinet, shelf, pantry, closet
**curtain**	rideau, rideaux, draper, draperie, store, jalousie	Curtain, curtains, drapery, drapes, blind
**dishcloth**	torchon, lavette, serviette, essuie-mains, essuie-verses, chiffon	towel, rag, napkin, wipe, cloth
**dish**	plat, assiette, tasse, coupe, gobelet, comptoir, vaisselle	plate, dish, cup, teacup, mug, counter, tableware
**exterior**	Extérieur, dehors, air, cour, yard, jardin, parc, préau, arrière-cour, allée, chemin, ruelle, voie, arbre, buisson, arbuste	outside, outdoors, air, courtyard, yard, garden, park, front yard, backyard, driveway, path, street, sidewalk, tree, bush, shrub
**girl**	fille, copine, sœur, enfant, enfants	girl, daughter, child, kid, school girl
**jar**	pot, bocal, jarre, récipient, conteneur, barquette, cruche, chope, boîte	pot, bowl, jar, receptacle, container, jug, box
**kitchen**	cuisine, chambre, salle	gallery, kitchenette, room, kitchen
**plate**	assiette, plaque	plate, platter, dish, saucer
**sink**	évier, lavabo, bassin, cuvette, lave-mains, robinet, taraud	sink, wash bowl, bassin, bowl, faucet, tap, spigot
**stool**	tabouret, selle, escabeau, banquette, siège, fauteuil, place, chaise, échelle, maille	footstool, chair, stepladder, seat, ladder
**water**	eau, eaux, lavasse, liquide, l'eau	water, flood, dish water, liquid
**window**	fenêtre, vitre, guichet, vitrine, cadre, encadrement, verre, miroir	window, pane, window pane, window frame, glass
**woman**	femme, femelle, adulte, dame, mère, maman, ménagère	wife, mother, mom, lady, adult, woman, female
